# Lactoferrin infant feeding trial_Canada (LIFT_Canada): protocol for a randomized trial of adding lactoferrin to feeds of very-low-birth-weight preterm infants

**DOI:** 10.1186/s12887-020-1938-0

**Published:** 2020-01-29

**Authors:** Elizabeth V. Asztalos, Keith Barrington, Abhay Lodha, William Tarnow-Mordi, Andrew Martin

**Affiliations:** 1 Department of Newborn and Developmental Paediatrics, Sunnybrook Research Institute, Sunnybrook Health Sciences Centre, University of Toronto, M4-230, 2075 Bayview Ave., Toronto, ON M4N 3M5 Canada; 2 Department of Pediatrics, Université de Montréal, Montréal, PQ Canada; 3 Department of Pediatrics, University of Calgary, Calgary, AB Canada; 4 NHMRC Clinical Trials Centre, University of Sydney, Sydney, Australia

**Keywords:** Very low birth weight infants, Lactoferrin, Neonatal mortality and morbidity

## Abstract

**Background:**

In Canada alone, almost 3000 VLBW infants are born and treated annually with almost 1200 going onto death or survival with severe brain injury, chronic lung disorders, aggressive retinopathy of prematurity, late-onset sepsis, or significant necrotizing enterocolitis. Lactoferrin is an antimicrobial, antioxidant, anti-inflammatory iron-carrying, bifidogenic glycoprotein found in all vertebrates and in mammalian milk, leukocytes and exocrine secretions. Lactoferrin aids in creating an environment for growth of beneficial bacteria in the gut, thus reducing colonization with pathogenic bacteria. It is hypothesized that oral bovine lactoferrin (bLF), through its antimicrobial, antioxidant and anti-inflammatory properties, will reduce the rate of mortality or major morbidity in very low birth weight preterm infants.

**Method:**

Lactoferrin Infant Feeding Trial_Canada (LIFT_Canada) is a multi-centre, double-masked, randomized controlled trial with the aim to enroll 500 infants whose data will be combined with the data of the 1542 infants enrolled from Lactoferrin Infant Feeding Trial_Australia/New Zealand (LIFT_ANZ) in a pooled intention-to-treat analysis. Eligible infants will be randomized and allocated to one of two treatment groups: 1) a daily dose of 200 mg/kg bLF in breast/donor human milk or formula milk until 34 weeks corrected gestation or for a minimum of 2 weeks, whichever is longer, or until discharge home or transfer, if earlier; 2) no bLF with daily feeds. The primary outcome will be determined at 36 weeks corrected gestation for the presence of neonatal morbidity and at discharge for survival and treated retinopathy of prematurity. The duration of the trial is expected to be 36 months.

**Discussion:**

Currently, there continues to be no clear answer related to the benefit of bLF in reducing mortality or any or all of the significant neonatal morbidities in very low birth weight infants. LIFT_Canada is designed with the hope that the pooled results from Australia, New Zealand, and Canada may help to clarify the situation.

**Trial registration:**

Clinical Trials.Gov, Identifier: NCT03367013, Registered December 8, 2017.

## Background

Over 15 million infants are born preterm (< 37 weeks gestation worldwide [1]. In developed countries such as Canada, Australia and the United States, very low birth weight infants (VLBW < 1500 g) preterm infants account almost 1.5% of all births [2–4]. In Canada alone, almost 3000 VLBW infants are born and treated annually with almost 1200 going onto death or survival with severe brain injury, chronic lung disorders, aggressive retinopathy of prematurity (ROP), late-onset sepsis (LOS), or significant necrotizing enterocolitis (NEC) [2]. Each of these morbidities has been associated with a substantial risk of childhood impairments [5–7].

Lactoferrin is an antimicrobial, antioxidant, anti-inflammatory iron-carrying, bifidogenic glycoprotein found in all vertebrates and in mammalian milk, leukocytes and exocrine secretions [8, 9]. Lactoferrin has been shown to be effective against infection when tested in animals and in the laboratory [10–12]. The systemic effects of oral lactoferrin are generally thought to be indirect and probably are initiated by contact with intestinal epithelial cells and gut-associated lymphoid tissue (GALT) [13, 14]. Lactoferrin and other similar products in human milk create an environment for growth of beneficial bacteria in the gut, reducing colonization with pathogenic bacteria. However, most VLBW infants receive insufficient human lactoferrin from human breast milk in the first months of life, resulting in suboptimal protection [15]. Because human lactoferrin is expensive, bovine lactoferrin has been considered as an alternate supplement to improve this suboptimal protection, as it has at least 70% amino acid homology with human lactoferrin with the same N-terminal peptide [16]. With the properties outlined, lactoferrin has emerged as a potential new tool for the prevention of serious neonatal morbidities.

There have been 6 randomized controlled trials involving almost 1100 preterm infants that have evaluated oral bovine lactoferrin (bLF) in the most recent Cochrane review (2017) [17].

The review showed that lactoferrin supplementation to enteral feeds decreased late-onset sepsis (typical risk ratio (RR) 0.59, 95% confidence interval (CI) 0.40 to 0.87; typical risk difference (RD) -0.06, 95% CI − 0.10 to − 0.02; and NEC stage II or III (typical RR 0.40, 95% CI 0.18 to 0.86; typical RD -0.04, 95% CI − 0.06 to − 0.01. Lactoferrin supplementation did not have an effect on “all-cause mortality” (typical RR 0.65, 95% CI 0.37 to 1.11; typical RD -0.02, 95% CI − 0.05 to 0). Lactoferrin supplementation to enteral feeds with or without probiotics decreased late-onset sepsis involving bacterial and fungal organisms but not chronic lung disease or length of hospital stay. Investigators reported no adverse effects and did not evaluate long-term neurological outcomes and periventricular leukomalacia. However, because of the moderate to low quality of the evidence as per the GRADE criteria [18], the Cochrane review called for the results of at least the 6 additional ongoing trials including LIFT_Canada to provide data to enhance the quality of the evidence.

### Collaboration with LIFT_Australia/New Zealand (LIFT_ANZ)

The Canadian-funded component of LIFT (LIFT_Canada) will enroll 500 participants to add to the 1542 participants already enrolled in LIFT_ANZ. By combining over 2000 recruited infants, the combined LIFT will have > 80% power to detect a 19% reduction in death or serious morbidity from a control rate of 26 to 19.5% at 2-tailed *p* < 0.05, which is clinically important, plausible, and more conservative than earlier trial results.

If LIFT confirms a 19% reduction in the RR of its primary outcome, bLF will have a major impact, translating into thousands more intact survivors without major morbidity in Australia, New Zealand, Canada, Europe and worldwide each year. As> 90% of very preterm survivors at hospital discharge reach adulthood [19, 20], this represents more than 19,000 life-years gained in Canada alone each year, one of the largest gains in intact survival - in any specialty - since neonatal surfactant and antenatal steroids [21, 22]. In addition, by following the infants to 24 months corrected age (CA), information on neurodevelopmental outcomes which is currently lacking will also be provided as survival without major morbidity has been associated with an improved chance of a positive neurodevelopmental outcome by 2 years corrected age [23].

### Rationale for continuing LIFT_Canada

Earlier in 2019, two large trials presented results, the Enteral Lactoferrin supplementation for very preterm infants (ELFIN trial) [24, 25] and Lactoferrin for Infant Feeding Trial_Australia New Zealand (LIFT_ANZ) [26].

In 2203 infants in the ELFIN Trial (2203 infants), lactoferrin was associated with a relative risk (risk ratio) for late onset sepsis of 0.95 (95% CI 0.86–1.04) [25]. This confidence interval did not reliably exclude a 14% reduction or a 4% increase in sepsis. In the 1417 infants in ELFIN who received formula for > 50% of days of enteral feeds, lactoferrin was associated with a relative risk for sepsis of 0.89 (95% CI 0.79–1.01). This did not reliably exclude a 21% reduction or 1% increase in sepsis in this subgroup. Overall, ELFIN did not rule out important potential benefits, particularly for infants who receive formula for over half of days of enteral feeds.

In 1542 infants in LIFT_ANZ, lactoferrin was associated with a relative risk for late onset sepsis of 0.82 (95% CI 0.63–1.07). LIFT_ ANZ did not rule out a 37% reduction or 7% increase in sepsis. (Presented at the 7th International Conference on Clinical Neonatology, Torino, Italy, May 2019).

When ELFIN is combined with LIFT_ANZ and all other trials, a total of 5011 infants, the overall effect of lactoferrin is to reduce the relative risk of late onset sepsis to 0.82 (95% 0.74–0.92; *P* = 0.0004), with moderate heterogeneity (I^2^ = 0.58) and asymmetric funnel plot, consistent with small study effects (personal communication, M Pammi).

Similarly, a meta-analysis of the effect of bLF on late onset sepsis in 1891 preterm infants who were not fed exclusively mother’s own milk in two RCTs of bLF supplementation [25, 27, 28], a relative risk of late onset sepsis to be reduced to 0.82 (95% 0.71–0.96; *P* = 0.01), with moderate heterogeneity (I^2^ = 0.64) and asymmetric funnel plot, consistent with small study effects, or true differences between trials in effectiveness or underlying patient risk [29].

This is consistent with a more moderate but clinically relevant average benefit in reducing sepsis with no evidence of harm and no clear increase in mortality, NEC, sepsis or other adverse outcomes. Consequently, a decision to continue enrollment has been supported by the Steering Committee for LIFT_Canada and LIFT_ANZ.

### Aim

Our primary hypothesis is that oral bovine lactoferrin (bLF), through its antimicrobial, antioxidant and anti-inflammatory properties, will reduce the rate of mortality or major morbidity in very low birth weight (VLBW) preterm infants. Thus our aim is two-fold: 1) to test the hypothesis that adding bLF to feeds in preterm babies of less than 1500 g birth weight will (i) improve survival free from major hospital morbidity (primary composite endpoint); and, (ii) have a beneficial effect on each component of the composite primary endpoint, as well as the number of blood transfusions given, length of hospital stay, time to full enteral feeds and growth to 36 weeks corrected gestation (CG); and 2) to evaluate the effect of bLF on survival and developmental outcomes at 24 months corrected age (CA).

## Methods

### Research questions

#### Primary research question

The primary research question: in very low birth weight preterm infants (< 1500 g), does the administration of bLF (200 mg/kg/day) to feeds daily compared to no bLF with feeds daily increase of decrease hospital mortality or major morbidity (defined as brain injury, late onset sepsis (LOS), necrotizing enterocolitis(NEC) at 36 weeks CG or retinopathy of prematurity (ROP) treated before discharge?

#### Secondary research questions

There are several secondary research questions. In very low birth weight infants, does giving bLF (200 mg/kg/day) increase or decrease:

(i) incidence of all-cause in-hospital mortality;
(ii) incidence of each of the 5 components of the composite primary outcomes;
(iii) chronic lung disease at 36 weeks CG
(iv) time to first day of full enteral feeds (≥120 ml/kg/day for 3 consecutive days);
(v) number of blood transfusions during the hospital stay;
(vi) length of initial hospital stay;
(vii) growth at 36 weeks CG as measured by weight and head circumference;
(viii) the incidence of death by 24 months CA or the presence of major neurodevelopmental outcomes at 24 months CA, as defined: (i) visual (cannot fixate/ legally blind, or corrected acuity < 6/60 in both eyes), or hearing impairment (requiring a hearing aid or cochlear implants); (ii) cerebral palsy with an inability to walk unassisted; (iii) major developmental delay involving cognition or speech (composite score < 85 for cognition or language on assessment)

### Design

**Fig. 1:**
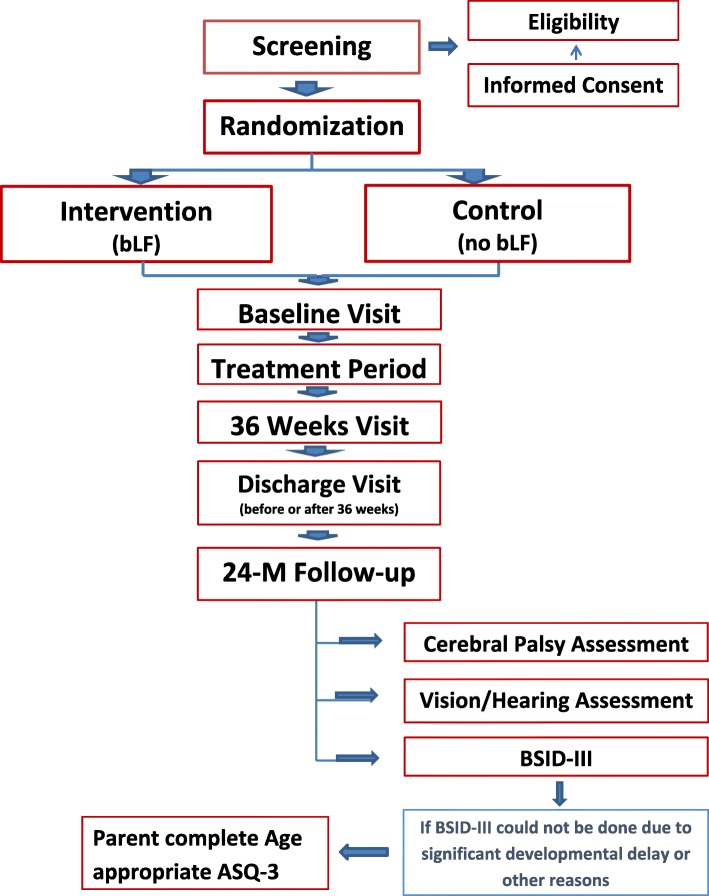
STUDY FLOW CHART

LIFT_Canada is a multi-centre, double-masked, randomized controlled trial with an intention-to-treat analysis. The aim is to enroll 500 infants whose data will be combined with the data of the 1542 infants enrolled from LIFT_ANZ in a pooled analysis (see Fig. 1 for study flow). Eligible infants will be randomized and allocated to one of two treatment groups: 1) a daily dose of 200 mg/kg bLF in breast/donor human milk or formula milk until 34 weeks CG or for a minimum of 2 weeks, whichever is longer, or until discharge home or transfer, if earlier; 2) no bLF with daily feeds.

The primary outcome will be determined at 36 weeks CG for the presence of neonatal morbidity and at discharge for survival and treated ROP.

At 24 months CA (with a window of ±6 months, i.e. 18–30 months CA), infants will be assessed with a neurodevelopmental assessment and information related to the status of vision, hearing, and cerebral palsy.

### Randomisation

Upon consent and confirmation of eligibility, the infant will be randomized using a 24-h/day web-based randomization service at the data coordinating centre at the NHMRC Clinical Trials Center in Sydney Australia. A study number will be issued. Study allocation will be randomly allocated in a 1:1 ratio using a minimization approach that stratifies for centre, gender, birth weight (< 1000 vs. ≥1000–1499 g) and according to whether the infant is from a multiple birth.

### Study setting

The study setting is national involving 6–8 centres (level 3 Neonatal Intensive Care Units) in Canada. (Table 1) Enrollment began in February 2018 and is expected to be completed in 36 months.

**Table 1:** List of Participating Centres (as of January 10, 2020)

Site	Name of REB	REB Number
BC Children’s Hospital	University of British Columbia Children’s and Women’s Research Ethics Board	H18–00588
Health Sciences Centre	University of Manitoba Biomedical Research Ethics Board	HS21705 (B2018:037)
Saint Boniface Hospital	University of Manitoba Biomedical Research Ethics Board	HS21705 (B2018:037)
The Ottawa Hospital	Clinical Trials Ontario	0890
McMaster Children’s Hospital	Clinical Trials Ontario	0890
Mount Sinai Hospital	Clinical Trials Ontario	0890
Sunnybrook Health Sciences Center	Clinical Trials Ontario	0890
IWK Health Center	IWK Research Ethics Board	1,023,505

REB- Research Ethics Board

### Ethics, informed consent, and safety

Documented approval has been obtained from the Research Ethics Board at Sunnybrook Health Sciences Centre which also serves as the Board of Record. Documented approval has been obtained from the Research Ethics Board/Institutional Review Board of all participating centres prior to the start of the study. In addition because bLF is not approved for use in preterm infants, documented approval has been obtained from Health Canada. The study is also designed to conform to the International Conference on Harmonisation, E6: Good Clinical Practice guidelines, and institutional policies.

An interim analysis is planned at 250 infants at which time all adverse events related to the study treatment and any sudden unexpected adverse events reported to Health Canada will be reviewed by the Data Safety Monitoring Committee.

Because the study participants are preterm infants, written informed consent is being obtained from the parents of the infant; infants are not considered for eligibility until a consent has been obtained from one or both parents.

### Eligibility

*Inclusion criteria:*

- < 1500 g at birth
- 2–7 days old and not moribund
- infant is deemed stable by the clinical care team
- has initiated feeds

*Exclusion criteria:*

- severe congenital anomalies which are likely to cause death or known to contribute to an adverse neurodevelopmental outcome
- major congenital gastrointestinal anomalies which will prevent an early approach to feeding
- parents unable to provide informed consent

### Duration of study period

The daily study treatment will start as soon as possible after randomisation (within 2 days) and continue until 34 weeks CG +/− 5 days or 2 weeks, whichever is longer, or until discharge home or transfer, if earlier.

The study treatment will be discontinued prior to the pre-specified discontinuation criteria if any one of the following occurs:

i. if the infant dies during the study period
ii. parental/guardian refusal for ongoing participation with the protocol
iii. if the clinical care team determines the need to do so.

If the study treatment is discontinued, participation in the study will continue with the parental/guardian’s permission, and infants will be followed according to the study protocol.

### Study outcomes

#### Primary outcome

The primary outcome is a composite of.

Hospital mortality or major morbidity at 36 weeks CG defined as:

i. Brain injury on ultrasound
ii. Necrotizing enterocolitis (Bell stage II or higher)
iii. Late onset sepsis (≥ 72 h of life, culture proven), or

Retinopathy of prematurity treated according to local guidelines before discharge from hospital.

#### Secondary outcomes

The secondary outcomes include:

- Incidence of all-cause in-hospital mortality
- the incidence of each of the 5 components of the composite primary endpoint
- chronic lung disease at 36 weeks CG
- Time to first day of full enteral feeds (≥120 ml/kg/day for 3 consecutive days)
- number of blood transfusions
- length of hospital stay
- weight and head circumference at 36 weeks CG
- the incidence of death by 24 months CA or the presence of major neurodevelopmental outcomes at 24 months CA, as defined: (i) visual (cannot fixate/ legally blind, or corrected acuity < 6/60 in both eyes), or hearing impairment (requiring a hearing aid or cochlear implants); (ii) cerebral palsy with an inability to walk unassisted; (iii) major developmental delay involving cognition or speech (composite score < 85 for cognition or language on developmental assessment)

### Statistical analysis

Analysis sets are defined as the following:

- The intention-to-treat (ITT) population will comprise all randomized infants
- The Per Protocol population will comprise all randomized infants that receive at least one administration of their assigned treatment and who are not deemed ineligible on blinded clinical review
- A second Per Protocol population will comprise all randomized infants who received the minimum required administrations of 14 days of their assigned treatment and who are not deemed ineligible on blinded clinical review.
- The safety population will comprise all randomized infants who received at least one dose of the assigned treatment. Infants will be analysed according to the treatment they received for the purposes of the safety analysis.

An interim analysis of the primary composite endpoint and of survival to discharge using the Haybittle-Peto [28] approach in the first 250 infants enrolled in the study in Canada. The procedure involves evaluating the test statistic calculated under the null hypothesis against a boundary of 3 standard deviations (equivalent to χ^2^ = 9, with a *p*-value of 0.0027. This will have a negligible effect on the alpha level applicable at the final analysis.

A two sided alpha (significance level) of 5% will be applied to the analysis of the primary composite endpoint. There will be no adjustment for the interim analysis evaluated using the Haybittle-Peto boundary [30].

The key secondary endpoints for LIFT_Canada comprise the individual components of the primary composite (survival, brain injury, NEC, LOS, and ROP). *P*-values adjusted for the 5 comparisons performed for this set of endpoints will be derived using the Benjami-Hochberg procedure [31] with a family wise error rate of 5%. Results of other endpoints, subgroup, and sensitivity analyses will be interpreted in proper context and with due consideration of type 1 error.

The primary analysis in LIFT_Canada is a pooled analysis utilizing the individual data of infants recruited in Canada combined with the data of the infants recruited in the LIFT_ANZ trial. Safety analyses will be performed using the safety population. Non-safety analyses will be performed using the ITT population. Sensitivity analyses will be performed using the Per Protocol populations. In addition, information will be presented as Canadian data only and combined poled (Canada and ANZ) data.

The primary analysis will be a comparison between treatment groups on the proportion of infants experiencing the primary composite endpoint that is tested using a Wald test (using a χ^2^ distribution) from a log-binomial model fitted using generalized estimating equations to accommodate correlation of data between siblings from multiple births. If the log-binomial model does not converge, a logistic model will be used.

Secondary categorical endpoints will be analysed using the same modelling approach applied to the primary endpoint (i.e. a log-binomial model fitted using the generalized estimating equations to accommodate possible correlation of data between sibling from multiple births; if the log-binomial model does not converge, a logistic model will be used).

A descriptive analysis of adverse events related to the study treatment and those reported to Health Canada in the Canadian cohort will be conducted by treatment allocation. The event rate is expected to be low and an exact test would be the appropriate choice to perform any formal comparisons between the two groups in the proportion experiencing an event related to the study treatment.

Consistency of the treatment effect on the primary endpoint across subgroups will be tested by including a treatment-by-subgroup interaction term (along with the relevant main effect terms) as covariates in the analysis model.

The subgroups of interest are

- Birthweight < 1000 g versus 1000-1499 g
- ≤ 28 weeks versus > 28 weeks gestation at birth
- Randomized ≤72 h versus > 72 h from birth
- Those who received probiotics by 36 weeks CG (note: this is a post-baseline covariate)
- Those who received human milk (mother’s own milk or donor human milk) versus formula as initial enteral feeds (note: this is a baseline covariate)

Both human milk as initial feeds and ‘probiotic use by 36 weeks CG are co-variates; an unbiased evaluation of their role as an effect-modifier will be attempted.

Randomisation is stratified by gender, birth weight (< 1000 g vs ≥ 1000-1499 g), single versus multiple birth, and centre. Sensitivity of results obtained from the primary analysis of the primary endpoint to adjustment for stratification factors will be explored by including them as covariates in the model.

Finally, an overall treatment adherence adjusted estimate of the effect of bLF on (i) the primary composite endpoint, and (ii) survival to hospital discharge will be calculated.

## Discussion

Currently, there continues to be no clear answer related to the benefit of bLF in reducing mortality or any or all of the significant neonatal morbidities in very low birth weight infants. Pooled data suggests benefit in some outcomes, in particular late onset sepsis. Benefit may be augmented in conjunction with current aspects of care such as use of human milk and probiotics in the vulnerable infants. It is hoped that the results of LIFT_Canada alone and pooled with LIFT_ANZ may help to clarify the situation.

## Data Availability

The datasets used and/or analysed for this study will be available from the corresponding author, NHMRC Clinical Trials Centre, and Sunnybrook Research Institute on reasonable request.

## Abbreviations

ANZ: Australia/New Zealand
bLF: Bovine lactoferrin
CA: Corrected Age
CG: Corrected Gestation
CI: Confidence interval
CIHR: Canadian Institutes for Health Research
ELFIN: Enteral lactoferrin supplementation for very preterm infants
GALT: Gut-associated lymphoid tissue
ITT: Intention-to-treat
LIFT: Lactoferrin Infant Feeding Trial
LOS: Late onset sepsis
NEC: Necrotising enterocolitis
NHMRC: National Health and Medical Research Council
RD: Risk difference
ROP: Retinopathy of prematurity
RR: Relative risk
VLBW: Very low birth weight
